# Freely available convolutional neural network-based quantification of PET/CT lesions is associated with survival in patients with lung cancer

**DOI:** 10.1186/s40658-022-00437-3

**Published:** 2022-02-03

**Authors:** Pablo Borrelli, José Luis Loaiza Góngora, Reza Kaboteh, Johannes Ulén, Olof Enqvist, Elin Trägårdh, Lars Edenbrandt

**Affiliations:** 1 Department of Clinical Physiology, Region Västra Götaland, Sahlgrenska University Hospital, Gothenburg, Sweden; 2 Eigenvision AB, Malmö, Sweden; 3 Department of Electrical Engineering, Chalmers University of Technology, Gothenburg, Sweden; 4 Department of Translational Medicine and Wallenberg Centre for Molecular Medicine, Lund University, Malmö, Sweden; 5 Department of Clinical Physiology and Nuclear Medicine, Skåne University Hospital, Malmö, Sweden; 6 Department of Molecular and Clinical Medicine, Institute of Medicine, Sahlgrenska Academy, University of Gothenburg, Gothenburg, Sweden

**Keywords:** Computer-assisted analysis, Tumour burden, Total lesion glycolysis, Prognosis

## Abstract

**Background:**

Metabolic positron emission tomography/computed tomography (PET/CT) parameters describing tumour activity contain valuable prognostic information, but to perform the measurements manually leads to both intra- and inter-reader variability and is too time-consuming in clinical practice. The use of modern artificial intelligence-based methods offers new possibilities for automated and objective image analysis of PET/CT data.

**Purpose:**

We aimed to train a convolutional neural network (CNN) to segment and quantify tumour burden in [^18^F]-fluorodeoxyglucose (FDG) PET/CT images and to evaluate the association between CNN-based measurements and overall survival (OS) in patients with lung cancer. A secondary aim was to make the method available to other researchers.

**Methods:**

A total of 320 consecutive patients referred for FDG PET/CT due to suspected lung cancer were retrospectively selected for this study. Two nuclear medicine specialists manually segmented abnormal FDG uptake in all of the PET/CT studies. One-third of the patients were assigned to a test group. Survival data were collected for this group. The CNN was trained to segment lung tumours and thoracic lymph nodes. Total lesion glycolysis (TLG) was calculated from the CNN-based and manual segmentations. Associations between TLG and OS were investigated using a univariate Cox proportional hazards regression model.

**Results:**

The test group comprised 106 patients (median age, 76 years (IQR 61–79); *n* = 59 female). Both CNN-based TLG (hazard ratio 1.64, 95% confidence interval 1.21–2.21; *p* = 0.001) and manual TLG (hazard ratio 1.54, 95% confidence interval 1.14–2.07; *p* = 0.004) estimations were significantly associated with OS.

**Conclusion:**

Fully automated CNN-based TLG measurements of PET/CT data showed were significantly associated with OS in patients with lung cancer. This type of measurement may be of value for the management of future patients with lung cancer. The CNN is publicly available for research purposes.

## Introduction

[^18^F]fluorodeoxyglucose (FDG) positron emission tomography/computed tomography (PET/CT) plays an important role in lung cancer, both for small cell and non-small cell cancer diagnosis, staging, response assessment and follow-up [1–4]. Several studies have shown the prognostic value of different metabolic PET parameters [5–10]. The methods to quantify tumour burden are, however, usually based on manually selected lesions by local imaging experts and easily accessible measurements such as maximum or peak standardized uptake value (SUV). An objective way to analyse the PET/CT findings would enable effectively comparing the results from different studies and, thereby, facilitate the assessment of the FDG PET/CT findings in patients with suspected lung cancer. The use of modern artificial intelligence (AI)-based methods offers new possibilities for automated and objective image analysis [11]. AI-based technology can be trained to assess the entire burden of disease by including both the extent and activity of the tumour and not only the maximum or peak SUV, which represents a very small volume of the tumour [12]. Manual methods of assessing total tumour burden, for example, the segmentation of all tumour lesions and estimation of total lesion glycolysis (TLG), are too time-consuming for clinical use and hampered by low reproducibility.

We recently trained a convolutional neural network (CNN) to automatically detect lesions and calculate the TLG from the FDG PET/CT data of patients with lung cancer [13]. That CNN has a sensitivity of 90% and the correlation between the manual and CNN-based automated TLG measurements is strong (r^2^ = 0.74). These results inspired us to take the next step and train a new CNN using a larger training set and expanding the task of the CNN to segment also thoracic lymph nodes. In addition, a new test group was selected in which survival data were available. Therefore, this study aimed to evaluate this new CNN by comparing its automated TLG measurements to corresponding manual measurements and by assessing the association between the TLG measurements and overall survival (OS) in patients with lung cancer. A secondary aim was to make the AI-based method freely available to other researchers.

## Methods

### Patients

Two groups of consecutive patients referred for FDG PET/CT due to suspected lung cancer were retrospectively selected to develop and evaluate the new CNN. One group of 113 patients underwent PET/CT between April 2008 and December 2010 at the Sahlgrenska University Hospital, Gothenburg, Sweden. This group was used in our first study to train and evaluate a CNN for the detection of lung tumours [13]. The other group of 207 patients underwent PET/CT between November 2017 and November 2018 at the Skåne University Hospital in Lund/Malmö, Sweden.

The total study group of 320 patients was divided into a test group of 106 patients (33%) and a training group of 214 patients (67%). Only patients from the Skåne University Hospital were selected randomly for the test group since the patients from the Sahlgrenska University Hospital were already used to train and test the CNN developed in our previous study. Clinical information and survival data for the test group were collected from local medical records and the radiology information system up until November 2020. The patient characteristics of the test group are presented in Table 1.

**Table 1:** Characteristics of the patients in the test group

	*n*	Median years (IQR)
Age	106	76 (61–79)
Sex		
Female	59	
Male	47	
Survival status		
Dead—survival time	51	0.9 (0.54–1.54)
Alive—follow-up time	55	2.6 (2.5–2.7)
Diagnosis		
Non-small cell lung carcinoma	85	
Lung cancer of unknown type	11	
Lung metastases	5	
Hamartoma	1	
Lymphoma	1	
Pneumoconiosis	1	
Schwannoma	1	
Unknown	1	

This study was conducted according to the principles expressed in the Declaration of Helsinki and approved by the local research ethics committees at Gothenburg (#295–08) and Lund Universities (#2016/193 and #2018/753). All patients provided written informed consent.

### Imaging

PET/CT scans were obtained using integrated PET/CT systems (Siemens Biograph 64 Truepoint, Siemens Healthineers, Erlangen, Germany and GE Discovery MI, GE Healthcare, Chicago, USA). The patients were injected with 4 MBq/kg (maximum of 400 Mbq) of FDG, fasted for at least 4 h prior to the injection and had adequate glucose levels prior to the injection. The accumulation time was 60 min. Images were acquired at 3 min per bed position (Sahlgrenska) or 1.5 min per bed position (Skåne) from the base of the skull to the mid-thigh.

PET images obtained from the Siemens Biograph 64 Truepoint PET/CT scanner were reconstructed with a slice thickness of 3 mm using an iterative ordered subset expectation maximization 3D algorithm (four iterations, eight subsets) with a matrix size of 168 × 168. CT-based attenuation and scatter corrections were applied. A low-dose CT scan (64-slice helical, 120 kV, 30 mAs, 512 × 512 matrix) was obtained covering the same area of the patient as the PET scan. The CT was reconstructed using a filtered back-projection algorithm with slice thickness and spacing that matched the PET scan.

The PET images obtained from the GE Discovery MI system were reconstructed using the commercially available block-sequential regularized expectation maximization (BSREM) algorithm Q.Clear (GE Healthcare, Milwaukee, WI, USA) with a beta factor of 550. The time-of-flight and point spread functions were used with a 256 × 256 matrix (pixel size 2.7 × 2.7 mm2, slice thickness 2.8 mm). CT images were acquired for attenuation correction and anatomical correlation of the PET images. A diagnostic CT with intravenous and oral contrast medium or a low-dose CT without contrast was performed. In our clinical routine, a low-dose CT is performed if a previous diagnostic CT was performed within 4 weeks. For diagnostic CTs, tube current modulation was applied by adjusting the tube current for each individual with a noise index of 42.25 and a tube voltage of 100 kV. For the low-dose CT, the tube voltage was 120 kV with a noise index of 45. If a diagnostic CT was performed, it was used for attenuation correction (delayed venous phase of intravenous contrast). The adaptive statistical iterative reconstruction technique (ASiR-V) was applied for all CT reconstructions.

### Manual segmentations

Two nuclear medicine specialists with over 6 and over 12 years of PET/CT experience segmented abnormal FDG uptake in the PET/CT images from the training and test groups. The segmentations were made manually by visual inspection in a consensus reading. Abnormal uptakes were classified into one of the following groups: lung tumour, thoracic lymph node, extra-thoracic lymph node, adrenal, bone, liver metastasis, inflammatory, high pleura or other high activity. No clinical data, only PET/CT images were available during the segmentation process. A cloud-based annotation tool (RECOMIA, https://www.recomia.org) was used for the manual segmentations [14].

### Convolutional neural network

The CNN was trained to segment lung tumours and thoracic lymph nodes only. The numbers of other abnormal uptake examples were insufficient for CNN training.

This model uses a CNN with U-net 3D architecture [15]. The final convolutional layer contains three channels with softmax activation, one for background, one for lung tumour and one for the thoracic lymph node. The network has three separate inputs, the CT image, the PET image and a one-hot encoded organ mask constructed using the model from [14]. The purpose of the organ mask is to help the network with a rough anatomical localization for a given uptake; it uses one channel each for bone, liver, lung, heart, aorta and adrenal gland.

The model was trained using patches of minimal size, where the patches were chosen with care to provide a good balance between the different classes. All pixels were divided into four groups: background, lung tumour, thoracic lymph node and other abnormal uptake (this group included extra-thoracic lymph nodes, metastases, inflammatory uptake, high pleura uptake and other high uptake). The centre point of each patch was chosen randomly with an equal probability of being inside any of the four groups.

The training involved 100 epochs with 10,000 patches per epoch. Categorical cross-entropy was used as the loss function, and the optimization was performed using the Adam method [16] with Nesterov momentum. In order to reduce overfitting, both early stopping (patience 10 epochs with no validation loss decrease) and l2 regularization with weight 0.01 were used. As pre-processing the CT image is clamped to HU range [− 800, 800] and the SUV image to [0, 25], both the CT and SUV images were then rescaled [− 1, 1]. The input patches were augmented using rotations (− 0.15 to 0.15) radians, scaling (− 10 to 10%) and intensity shifts of (− 100 to + 100HU) for the CT images and (− 0.5 to + 0.5) for the SUV image. After this phase, the resulting model was applied to the training set. The model was then retrained with 20% of the patches focusing on pixels incorrectly classified by the model. These steps were repeated four times. The resulting model is the last model after these four steps and early stopping (not the model with lowest validation loss). Finally, lung tumours and thoracic lymph nodes with TLGs below 0.1 were removed.

### Statistical analysis

Associations between TLG and OS were investigated using a univariate Cox proportional hazards regression model. OS was calculated from the date of the PET/CT analysis to the date of death or the last follow-up. Hazard ratios (HRs) and 95% confidence intervals (CIs) were estimated. The level of significance was set at 0.05. The TLG measurements had a skewed distribution and were log10-transformed after adding 1.0 to handle any zeros. The TLG measurements for the Kaplan–Meier analysis were categorized according to the corresponding median value, higher vs. lower than the median. The statistical analysis was performed in R (version 4.0.3) [17].

## Results

### TLG measurements

**Fig. 1:**
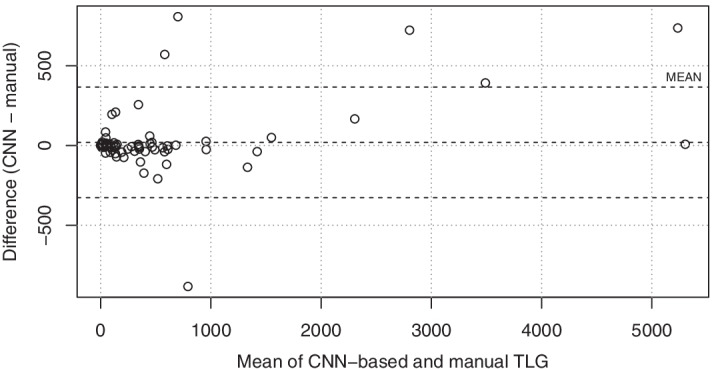
Bland–Altman plot comparing CNN-based and manual TLG measurements

**Fig. 2:**
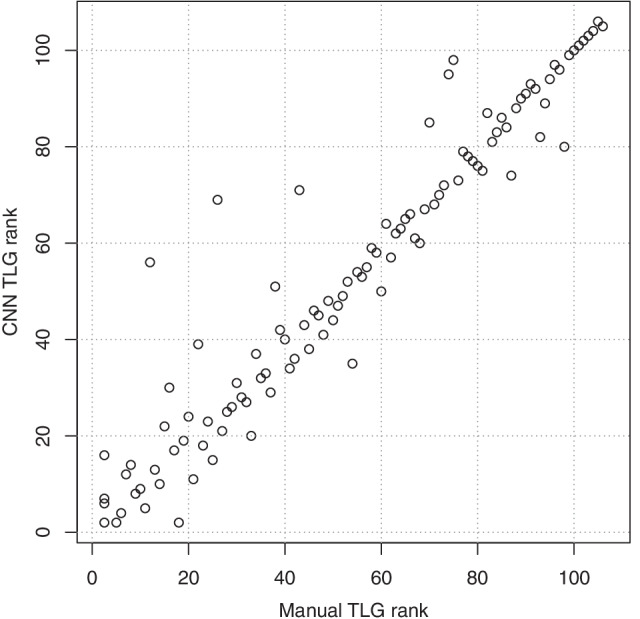
The rank of each study based on manual TLG measurements against the rank of each study based on the CNN-based measurements. This serves to show, that in terms of ranking patients with respect to tumour burden the automated method is similar to a manual analysis

Uptake from lung tumours and thoracic lymph nodes were used to compute a total TLG for each patient, both from the CNN and manual segmentations. Figure 1 shows a Bland–Altman plot comparing the CNN and manual TLG. Figure 2 plots the rank of each study based on manual TLG against the rank based on CNN-based TLG. The Spearman correlation of the TLG measurements, being the Pearson correlation of these rank values, is 0.95.

Both CNN TLG (HR 1.64, 95% CI 1.21–2.21; *p* = 0.001) and manual TLG (HR 1.54, 95% CI 1.14–2.07; *p* = 0.004) were significantly associated with OS in univariate proportional regression Cox analyses.

**Fig. 3:**
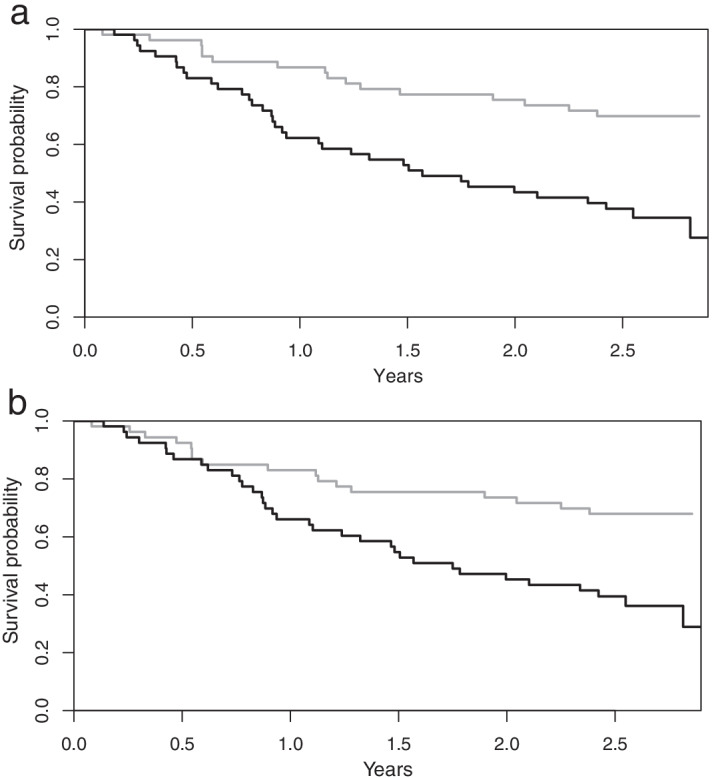
Kaplan–Meier curves for the groups higher (black line) vs. lower (grey line) than the median TLG values for CNN TLG (**a**) and manual TLG (**b**)

The Kaplan–Meier curves are shown in Fig. 3. The 53 patients with CNN TLGs above the median value had a significantly shorter survival time than the 53 patients with values below the median. The median survival times for the two groups were 1.57 years vs. not reached after 3 years of follow-up. The log-rank test showed a significant difference in survival times (*p* < 0.001). The corresponding median survival values for the two groups stratified using manual TLG were 1.75 years vs. not reached after 3 years of follow-up (*p* = 0.002).

### Lesion classification results

The CNN and the physicians classified 99/106 (93%) patients similarly regarding the presence of a lung tumour, 94 as positive and 5 as negative. Four patients were classified as positive only by the CNN. The CNN detections in these four cases were classified as thoracic lymph nodes (2), high pleura uptake (1) and inflammatory uptake (1) by the physicians. Three patients were classified as positive only by the physicians. A total of 135 lung tumours were detected by both the CNN and the physicians. These tumours had a median TLG of 13.9 (IQR 2.3–181.7). A total of 56 lesions with a median TLG of 0.4 (IQR 0.1–1.6) were detected as lung tumours by the physicians only. Seven of these lesions were classified as thoracic lymph nodes by the CNN. A total of 153 lesions with a median TLG of 0.6 (IQR 0.3–2.3) were detected as lung tumours by the CNN only. Thirty-five of these lesions were classified as thoracic lymph nodes by the physicians.

The CNN and the physicians classified 71/106 (67%) patients similarly regarding the presence of thoracic lymph nodes, 59 as positive and 12 as negative. Thirty-five patients were classified as positive only by the CNN. Both the CNN and the physicians detected 170 thoracic lymph nodes. These lesions had a median TLG of 5.8 (IQR 1.5–27.9). A total of 70 lesions with a median TLG of 0.3 (IQR 0.1–0.9) were detected as thoracic lymph nodes by the physicians only. Five of these lesions were classified as lung tumours by the CNN. A total of 274 lesions with a median TLG of 0.8 (IQR 0.3–2.4) were detected as thoracic lymph nodes by the CNN only. Thirty-two of these lesions were classified as lung tumours and 23 as extra-thoracic lymph nodes by the physicians.

## Discussion

In this study, we explored if automated TLG measurements calculated by a CNN were clinically relevant in patients with lung cancer. The results show that a CNN can be trained to automatically segment lung tumours and thoracic lymph nodes and calculate TLG measurements that are significantly associated with OS. The CNN TLG had a similar prognostic performance as the corresponding manual measurements.

In a clinical setting, a physician would be able to check CNN-based segmentations and dismiss false-positive lesions. The use of AI support for the calculation of total lesion uptake significantly reduces inter-reader variability in the analysis of prostate lesions and bone metastases with PSMA PET/CT [18]. This process is also more time-effective than a completely manual segmentation process and feasible for a clinical setting.

In research, these types of AI tools may facilitate comparisons between studies from different centres, pooling data within multicentre trials and performing meta-analyses when objective evaluation is applied rather than local image readers.

Objective AI-based TLG measurements may be useful not only for the prognostic evaluation of patients with lung cancer at the time of diagnosis but also for monitoring treatment response. FDG PET/CT findings have been associated with clinical benefit in patients with non-small cell lung cancer receiving immunotherapy [4].

AI tools have been used as computer-aided detection (CAD) systems to highlight potential lesions in chest X-rays and lung CTs [19]. The aim is to decrease the likelihood of a radiologist missing tumours and the subsequent delay in diagnosis. Such an AI tool needs to have high sensitivity and a low false-positive rate to be of clinical value. The aim of our CNN was not to be a CAD system but a tool for the automated quantification of tumour burden. The sensitivity and false-positive rate of our CNN were not sufficient for use as a clinical CAD system. The disagreements between the physicians and the CNN were, in most cases, related to lesions with low TLG. A common reason for any disagreement was physicians classifying a lesion as a lung tumour and the CNN as a mediastinal/hilar lymph node or vice versa; this is not always an easy decision even for experienced physicians.

A limitation of this study is the reference method using the manual segmentations by two nuclear medicine specialists. A slightly different result would most likely have been found if other, additional image readers had been used given the well-known problems of inter- and intra-observer variability. The comparisons between the CNN-based and manual TLG measurements indicate that the CNN was trained to perform similarly to a nuclear medicine specialist. A strength of this study is that we also used OS as an image-independent reference method.

The retrospective design of this study is, on the one hand, a limitation of the study but allowed us to assess the performance of the CNN not only compared with manual segmentation of the same images but also to the independent reference OS. The training material only contained a handful of abnormal uptake other than lung tumours and thoracic lymph nodes, due to this we limited this work to these two uptakes. Extra-thoracic lymph nodes and distant metastases will be added in future versions of our CNN. Further development will also include the localization of lymph nodes into established anatomical definitions and classifications into ipsilateral versus contralateral regions [20]. This type of information may improve the prognostic value of AI-based analyses.

The development of AI-based analyses depends on the availability of large training and test groups. The training and test groups used in this study were sufficient to show the proof of concept of a fully automated AI-based PET/CT analysis, but we recognize that larger patient groups including patients from several hospitals would improve the CNN-based method and strengthen the results. One approach to achieve this is to invite others researchers to participate in the development and we therefore make our CNN available to others. Another approach is to use methods to maximize the utility of incomplete and missing data as presented by Guo and co-workers [21].

## Conclusions

Fully automated CNN-based TLG measurements of FDG PET/CT data were significantly associated with OS in patients with lung cancer. These types of measurements may be of value in the management of future patients with lung cancer. Our CNN is available for research purposes upon request from the RECOMIA platform (https://recomia.org).

## Data Availability

Our CNN is available for research purposes upon request at the RECOMIA platform (https://recomia.org). The datasets generated and analysed for the current study are not publicly available due to privacy restrictions from the hospitals providing them.
